# New Work on Obstetrics and Diseases of Women and Children

**Published:** 1847-08

**Authors:** Jno. Evans


ARTICLE IX.

### NEW WORK

### ON OBSTETRICS AND THE DISEASES PECULIAR TO WOMEN AND CHILDREN.

Having in process of preparation for the press a work on the subjects designated above, which is intended to be a compendious system of facts for the student and practitioner, and being desirous of rendering it more particularly useful to those of the great west, I take this method of soliciting the physicians of the western country to communicate to me the results of their observations upon the modifications that the diseases peculiar to women and children present, as they occur in their respective districts. Valuable aid of this kind shall be properly accredited in the body of the work.

JNO. EVANS.

